# Facile fabrication of one-dimensional Te/Cu_2_Te nanorod composites with improved thermoelectric power factor and low thermal conductivity

**DOI:** 10.1038/s41598-018-35713-9

**Published:** 2018-12-24

**Authors:** Dabin Park, Hyun Ju, Taeseob Oh, Jooheon Kim

**Affiliations:** School of Chemical Engineering & Materials Science, Chung-Ang University, Seoul, 06974 Republic of Korea

## Abstract

In this study, Te/Cu_2_Te nanorod composites were synthesized using various properties of Cu_2_Te, and their thermoelectric properties were investigated. The nanorods were synthesized through a solution phase mixing process, using polyvinylpyrrolidone (PVP). With increasing Cu_2_Te content, the composites exhibited a reduced Seebeck coefficient and enhanced electrical conductivity. These characteristic changes were due to the high electrical conductivity and low Seebeck coefficient of Cu_2_Te. The composite containing 30 wt.% of Cu_2_Te nanorods showed the maximum power factor (524.6 μV/K at room temperature). The two types of nanorods were assembled into a 1D nanostructure, and with this structure, thermal conductivity decreased owing to the strong phonon scattering effect. This nanorod composite had a dramatically improved *ZT* value of 0.3, which was ~545 times larger than that of pristine Te nanorods.

## Introduction

The global energy crisis and environmental problems caused by burning fossil fuels have drawn much attention to alternative energy sources. Thermoelectric (TE) energy conversion is highly attractive as a promising energy-harvesting strategy because it can be used to, directly generatie electrical energy from temperature gradients^1–3^. The dimensionless figure of merit (*ZT*) is used to evaluate the performance of TE materials; a large *ZT* is required to achieve a high energy conversion efficiency. *ZT* can be expressed as shown in Equation 1.

1 $$ZT={S}^{2}\sigma T/\kappa $$

In Equation 1, *S* is the Seebeck coefficient, *σ* is the electrical conductivity, *к* is the total thermal conductivity, and *T* is the absolute temperature. The thermoelectric performance of a material can be enhanced by two simple strategies: reducing its thermal conductivity, or enhancing its power factor (*PF* = *S*^2^*σ*), which can be accomplished by decoupling the relationship between *S* and *σ*.

An outstanding Seebeck coefficient is one of the most important prerequisites for obtaining a large power factor. The *S* value of a material is generally considered inversely proportional to its *η* (carrier concentration), as shown in the following relatively simple model of electron transport (Equation 2).

2 $${\rm{S}}=\frac{8\cdot {\pi }^{2}\cdot {{k}_{B}}^{2}}{3\cdot e\cdot {h}^{2}}\cdot {m}^{\ast }\cdot T\cdot {(\frac{\pi }{3\cdot \eta })}^{\frac{2}{3}}$$

In Equation 2, *h* is the Planck constant, *k*_*b*_ is the Boltzmann constant, and m* equals 0.58 *m*_*e*_, where *m*_*e*_ is the electron resting mass. From this equation, it can be seen that the *S* value increases as *η* decreases. In recent studies, inorganic materials have mainly been used as thermoelectric devices, due to their high Seebeck coefficients, which are caused by their crystalline structures. In recent years, Te and its alloys have shown great potential as efficient thermoelectric materials, because of their unique characteristics^4–6^. Te exhibits excellent thermoelectric performance, and has an extremely high Seebeck coefficient. (~400 μV/K at 300 K)^7^, which is higher than that of Te alloys such as Bi_2_Te_3_ (~170 μV/K)^8^, PbTe (~200 μV/K)^9^, Sb_2_Te_3_ (~160 μV/K)^10^, or Ag_2_Te (~90 μV/K)^11^. Despite this advantage, Te is not a perfect high power factor material, due to its electrical conductivity (~12 S/m at 300 K)^12^, which is lower than that of other Te alloys.

To solve this problem, some researchers have focused on improving the electrical conductivity of Te-based thermoelectric devices^13–16^. Preparing composites of Te with other similar materials is a simple way to improve its thermoelectric properties. One example is the polymer-Te hybrid composite of PANI and Te nanorods, which are reported to have a high *ZT* value of 0.23 at 383 K^17^. Zhang *et al*.^18^ prepared Bi_2_Te_3_-Te heterophase nanoparticles, which also achieved an enhanced power factor.

Among the various Te alloys, copper (Ι) telluride (Cu_2_Te) has shown relatively high electrical conductivity (~40,000 S/m at 300 K)^19^ when compared with other Te alloys - Ag_2_Te (~20,000 S/m at 300 K)^20^, Bi_2_Te_3_ (~22,000 S/m at 300 K)^21^, and pure Te – and was also reported to have an outstanding Seebeck coefficient. Additionally, reports of thermoelectric materials containing Cu_2_Te have been published in recent years, and so Cu_2_Te is considered to be an appropriate material for increasing the power factor of Te.

Low thermal conductivity is another important factor in achieving a high *ZT*. One-dimensional (1D) nanostructures have many unique physical and chemical properties, and are highly promising materials for various applications^22,23^. 1D nanostructures can reduce thermal conductivity by increasing phonon scattering. Materials with 1D nanostructure generate many phonon scattering sites, and scatter phonons more efficiency than bulk materials^24,25^, a phenomenon that reduces total thermal conductivity as a consequence. Nanostructured, Te-based thermoelectric materials, with lower thermal conductivity than the corresponding bulk counterparts, were discussed in our previous papers^26,27^. In addition, Alam *et al*. reported an enhancement of *ZT* by reducing the thermal conductivity on the basis of the nanostructure of the material^28^. Yang *et al*.^29^ obtained significantly reduced thermal conductivity using nanostructured Bi_2_Te_3_.

In this study, Te/Cu_2_Te nanorod composites made from Te and Cu_2_Te nanorods were fabricated to achieve improved thermoelectric properties. The Te nanorods were prepared using a polyvinylpyrrolidone (PVP)-assisted, solution-phase synthetic process, from a Te precursor solution. The role of PVP in this study was to restrict the growth direction to one dimension, and to control the growth rate. Cu_2_Te nanorods were synthesized from the fabricated Te nanorods by solution phase mixing. After the fabrication of both types of nanorods, the composites were fabricated via ultrasonication. The homogeneous dispersion of the two types of nanorods affected the intrinsic conduction of the composites and was thought to potentially enhance their thermoelectric properties.

On this basis, the thermoelectric properties of the Te/Cu_2_Te nanorod composite samples, with varied Cu_2_Te content, were investigated, and our hypothesis was that the combination of these two nanorods would affect each other and enhance their thermoelectric properties.

## Results and Discussion

**Figure 1:**
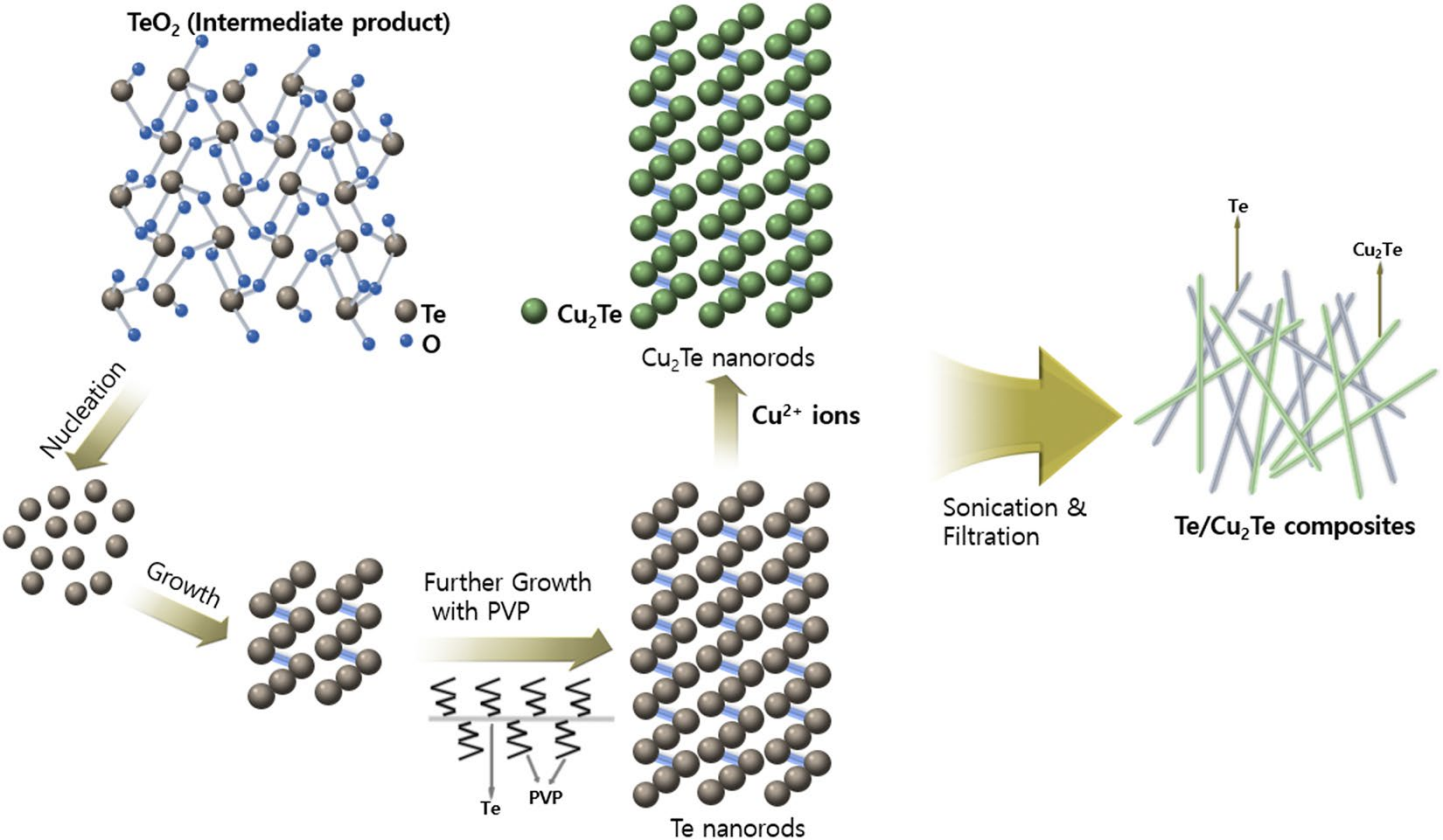
Schematic illustration of the fabrication of Te/Cu_2_Te nanorod composite.

Figure 1 shows the overall synthesis of the Te/Cu_2_Te nanorod composites. Both the Te and Cu_2_Te nanorods were prepared using a PVP-assisted, solution-phase mixing process. In the first stage of the Te nanorod synthesis, TeO_2_ was mixed with PVP and NaOH in ethylene glycol (EG). After the temperature was raised to 120 °C, N_2_H_4_·H_2_O was injected into the solution. The reaction process during the Te synthesis steps is shown in Equations 3–5.

3 $${{\rm{TeO}}}_{2}+{\rm{2NaOH}}\to {{{\rm{TeO}}}_{3}}^{2-}+{{\rm{2Na}}}^{+}+{{\rm{H}}}_{2}{\rm{O}}$$

4 $${{{\rm{TeO}}}_{3}}^{2-}+{{\rm{2Na}}}^{+}\to {{\rm{Na}}}_{2}{{\rm{TeO}}}_{3}$$

5 $${{\rm{Na}}}_{2}{{\rm{TeO}}}_{3}+{{\rm{N}}}_{2}{{\rm{H}}}_{4}\cdot {{\rm{H}}}_{2}{\rm{O}}\to {\rm{Te}}+{{\rm{N}}}_{2}+{\rm{2NaOH}}+2{{\rm{H}}}_{2}{\rm{O}}$$

The reaction process can be divided into two stages. First, tellurium dioxide reacts with NaOH to form TeO_3_^2−^ (Equation 3). Then, the generated TeO_3_^2−^, and two Na^+^ ions produce Na_2_TeO_3_. (Equation 2). Subsequently, hydrazine monohydrate reduces the Na_2_TeO_3_ to elemental Te (Equation 3). During this stage, the nucleation of Te^2−^ ions occurs, the elemental Te is formed with reduction of the Te^2−^ ions, and a solid crystal nucleus is formed. The growth of the Te crystal nuclei into Te nanorods was accelerated by reduction, and the concentration of Te^2−^ ions in the solution was decreased.

PVP, used as the surfactant, played an important role in the Te nanorod synthesis. It has been reported that linear polymers can react with inorganic ions to form chain-shaped intermediates^30,31^. In this study, PVP served as a directing template for the fabrication of the 1D nanostructure. In other words, PVP controlled the growth rate, and maintained the 1D growth direction, of the nanorods.

The Cu_2_Te nanorods used in this study were fabricated from the synthesized Te nanorods. The Cu precursor (Cu(NO_3_)_2_) was reacted with the Te nanorods to generate Cu_2_Te nanorods. The transformation of Te nanorods into Cu_2_Te nanorods involved the following reactions: First, the Cu^2+^ ions released by Cu(NO_3_)_2_ are absorbed on the surface of the Te nanorods. After ascorbic acid (a weak reducing agent) is injected into the mixture with the Te and Cu^2+^ ions, the Cu^2+^ ions are reduced to Cu^+^. Second, the reduced Cu+ ions induce an imbalanced response at the surface of the Te nanorods, by which Te → Te^4+^ + Te^2−^. Finally, the Cu^+^ ions react with Te^2−^, and Cu_2_Te nanorods are formed.

**Figure 2:**
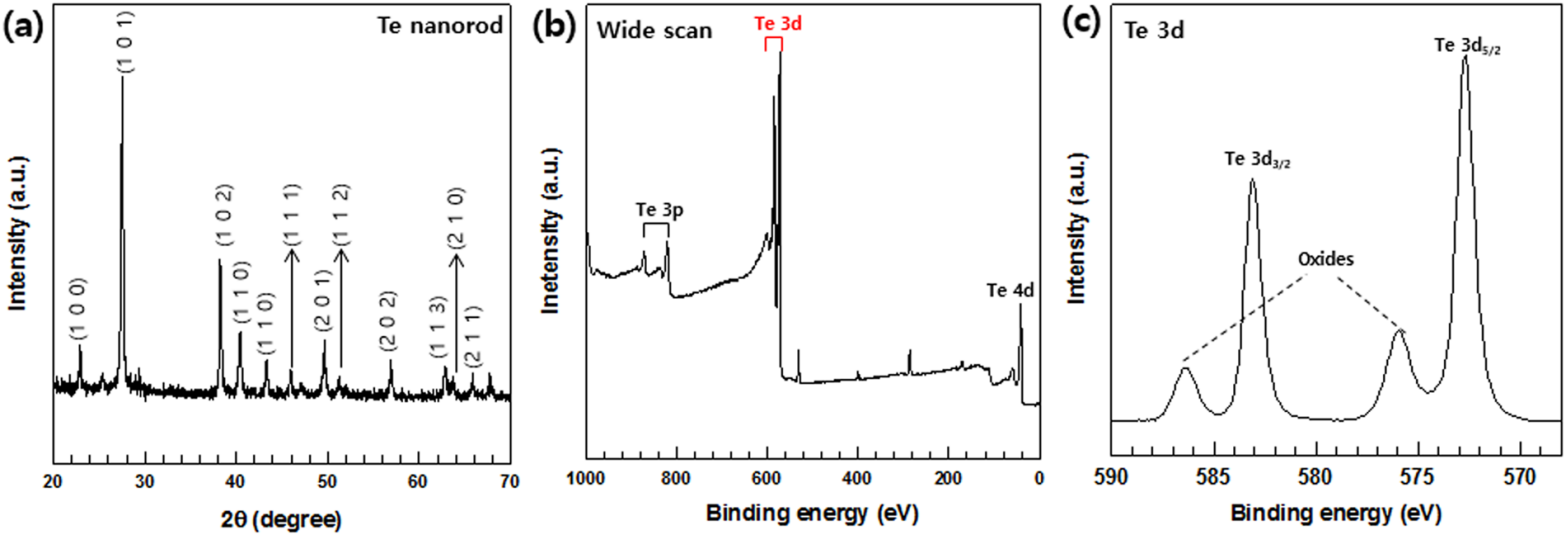
(**a**) XRD patterns, and (**b**,**c**) XPS spectra of Te nanorods: (**b**) Wide XPS scan for Te, (**c**) Te 3d spectrum.

**Figure 3:**
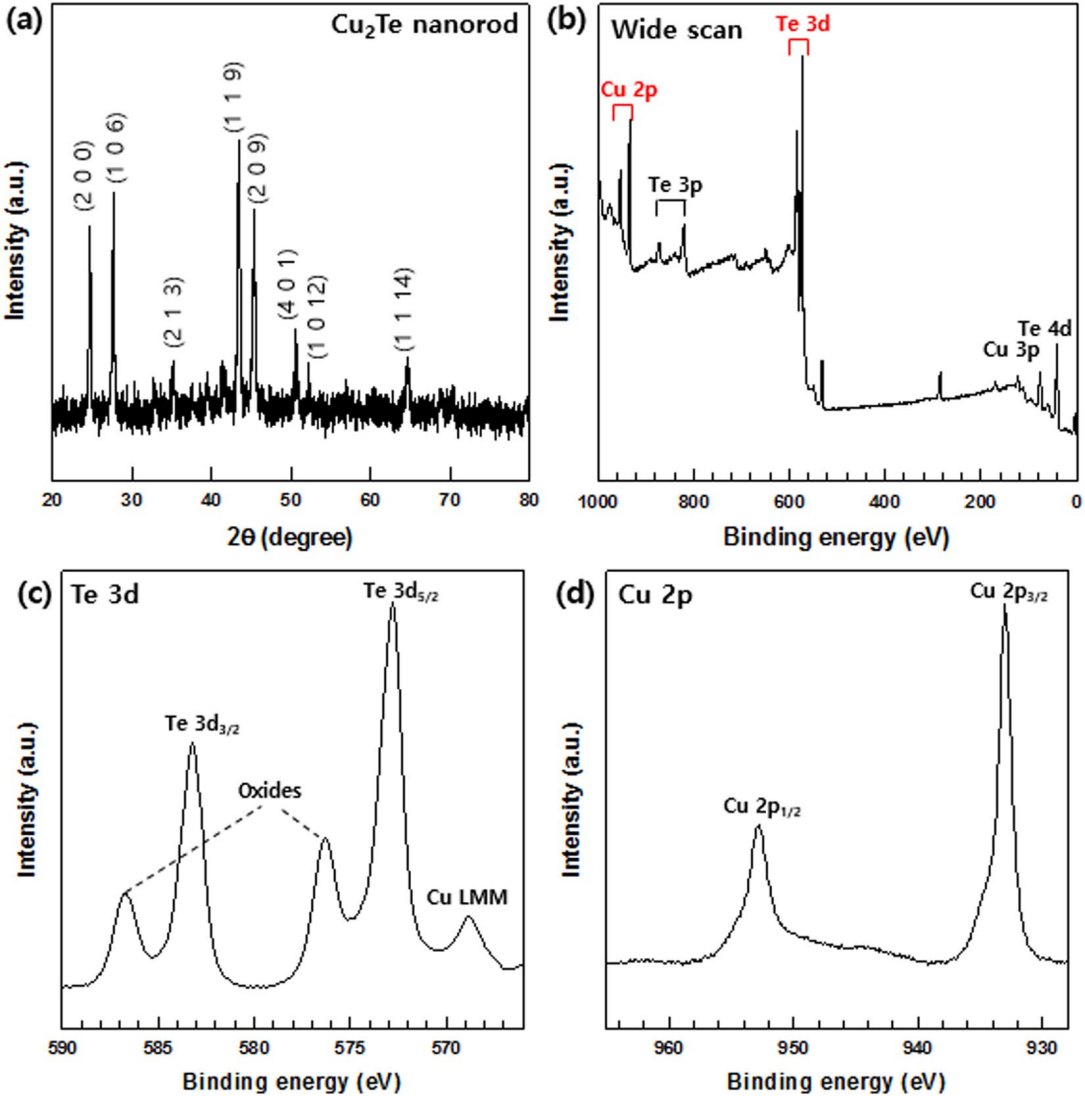
(**a**) XRD patterns, and (**b**–**d**) XPS spectra of Cu_2_Te nanorods: (**b**) Wide XPS scan for Cu_2_Te, (**c**) Te 3d spectrum, (**d**) Cu 2p spectrum.

XRD patterns of the two types of nanorods were measured to confirm their synthesis. The XRD patterns of the Te and Cu_2_ nanorods are shown in Figs 2a and 3a, respectively. This pattern indicated the hexagonal crystalline phase of the Te product and was in good agreement with the literature values (JCPDS no. 13-1452). In Fig. 3a, all diffraction peaks in the XRD pattern can be indexed to the hexagonal Cu_2_Te phase and are consistent with those reported in the literature (JCPDS no. 10-0421). No other peaks were observed in either of the two patterns, indicating the successful fabrication of pristine Te and Cu_2_Te nanorods.

The synthesis of the Te and Cu_2_Te nanorods was further confirmed using XPS analysis. The resulting XPS survey spectra for the prepared Te and Cu_2_Te nanorods are illustrated in Figs 2 and 3. A high-resolution spectrum of the Te 3d region (Fig. 2c) shows two peaks at approximately 572.5 and 582.9 eV: these two peaks correspond to the Te 3d_5/2_ and Te_3/2_ binding energies of Te. Two relatively small peaks can be seen at 576.1 and 586.4 eV, which can be assigned as the Te(IV) 3d binding energy, indicating the oxidation of Te. These peaks are observed because the surface of Te nanorods is easily oxidized in air.

Nanostructured materials are more easily oxidized in air, as has been reported in previous studies^32^. The XPS data of the Cu_2_Te nanorods are shown in Fig. 3b–d, in which the Cu 2p and Te 3d peaks are observed. The high-resolution spectra of the Te 3d and Cu 2p regions are shown in Fig. 3c,d, repectively. In the Cu 2p region, two peaks at 932.2 and 952.4 eV are observed, which correspond to Cu 2p_3/2_, and Cu 2p_1/2_, and the two peaks located at 589.2 eV and 572.5 eV in Fig. 3d correspond to the Te 3d_3/2_, and 3d_5/2_ binding energies. The Cu_2_Te nanorods are nanostructured materials, and thus are also easily oxidized in air. As a result, the oxidation peaks of Cu and Te are observed in Fig. 3c,d, respectively

**Figure 4:**
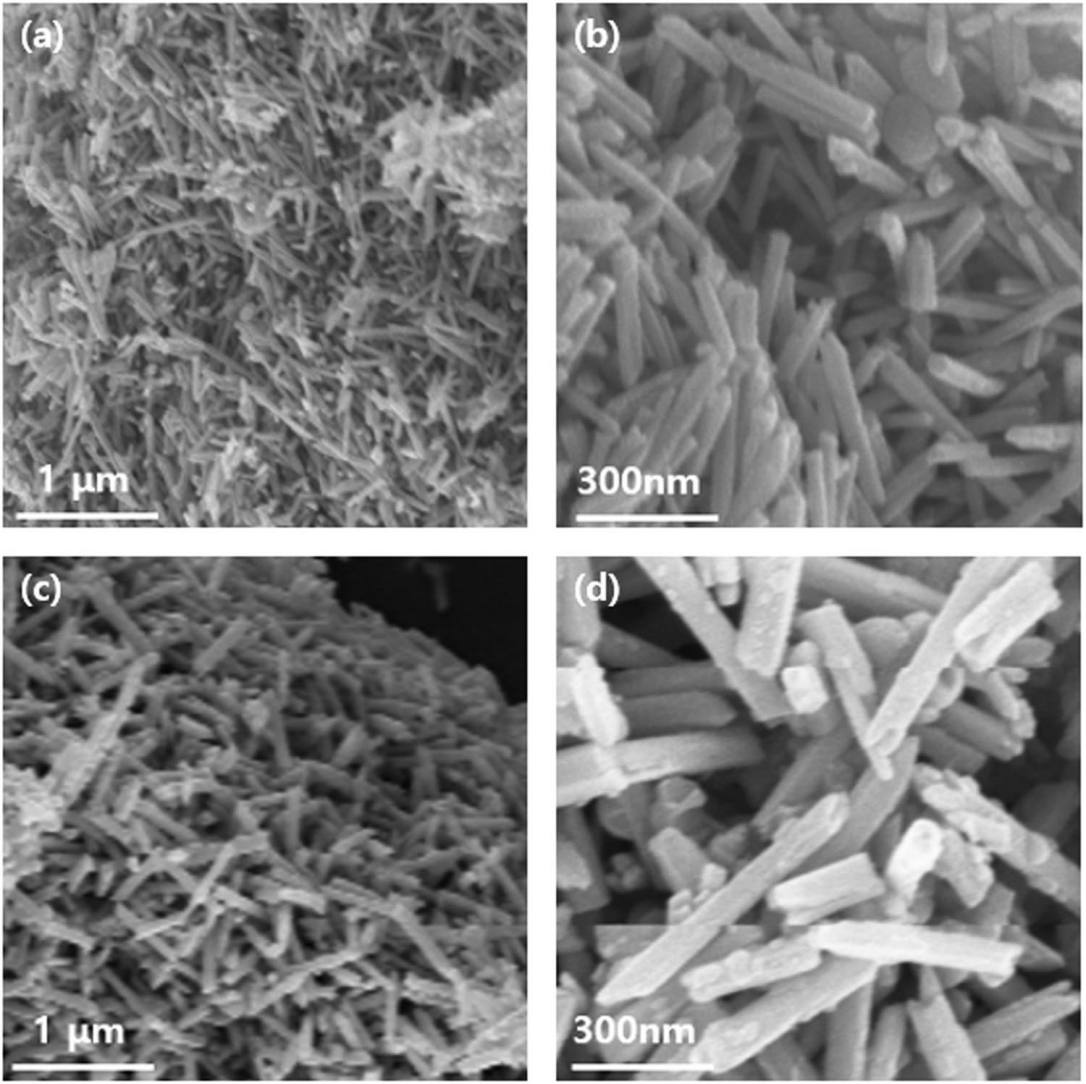
(**a**) Low and (**b**) high-magnification FE-SEM images of Te nanorods, (**c**) low and (**d**) high-magnification FE-SEM image of Cu_2_Te nanorods.

**Figure 5:**
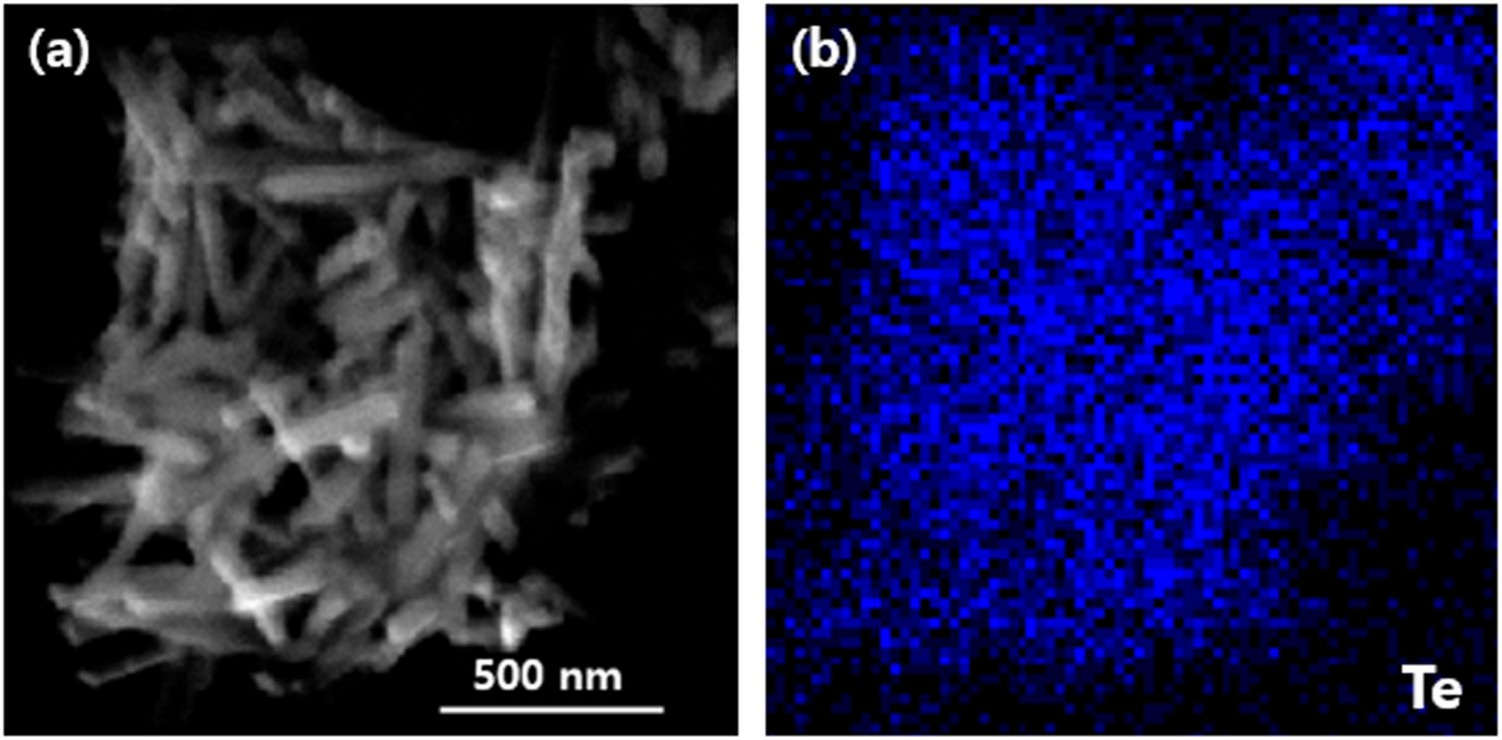
Particle surface morphology and atomic mapping of Te nanorods.

**Figure 6:**
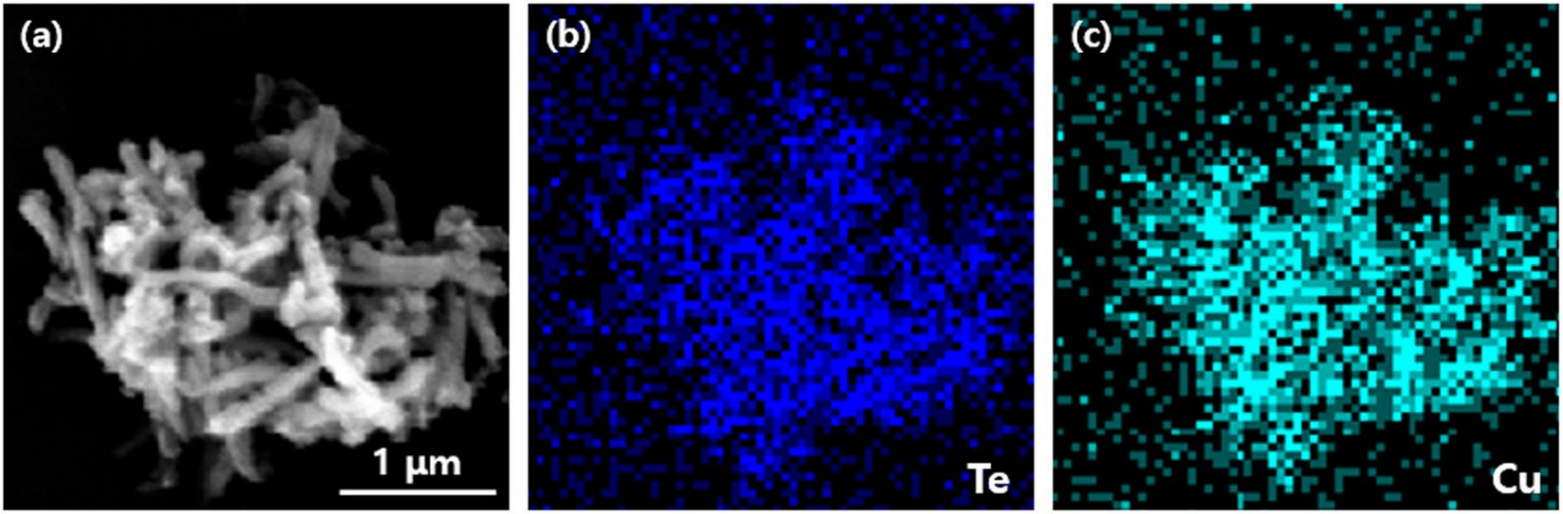
Particle surface morphology and atomic mapping of Cu_2_Te nanorods.

To confirm the 1D nanostructure, crystallinity, and the morphology of the synthesized nanorods, FE-SEM and EDS analyses were carried out. Low and high-resolution FE-SEM images of the fabricated Te nanorods are shown in Fig. 4a,b and demonstrate the existence of a large number of randomly dispersed wire-like structures. The obtained products consisted mainly of cylindrically shaped rods of a relatively uniform size. Each Te nanorod was ~15 nm in length, and ~600 nm in diameter. The images at Fig. 4c,d shows the presence of Cu_2_Te nanorods, confirming the successful synthesis of Cu_2_Te. These results were further verified using EDS mapping. Figs 5 and 6 illustrates the EDS atomic mapping of the Te and Cu_2_Te nanorods, respectively The EDS spectrum collected in the specific region highlighted in Fig. 6b,c indicates the presence of Cu and Te.

**Figure 7:**
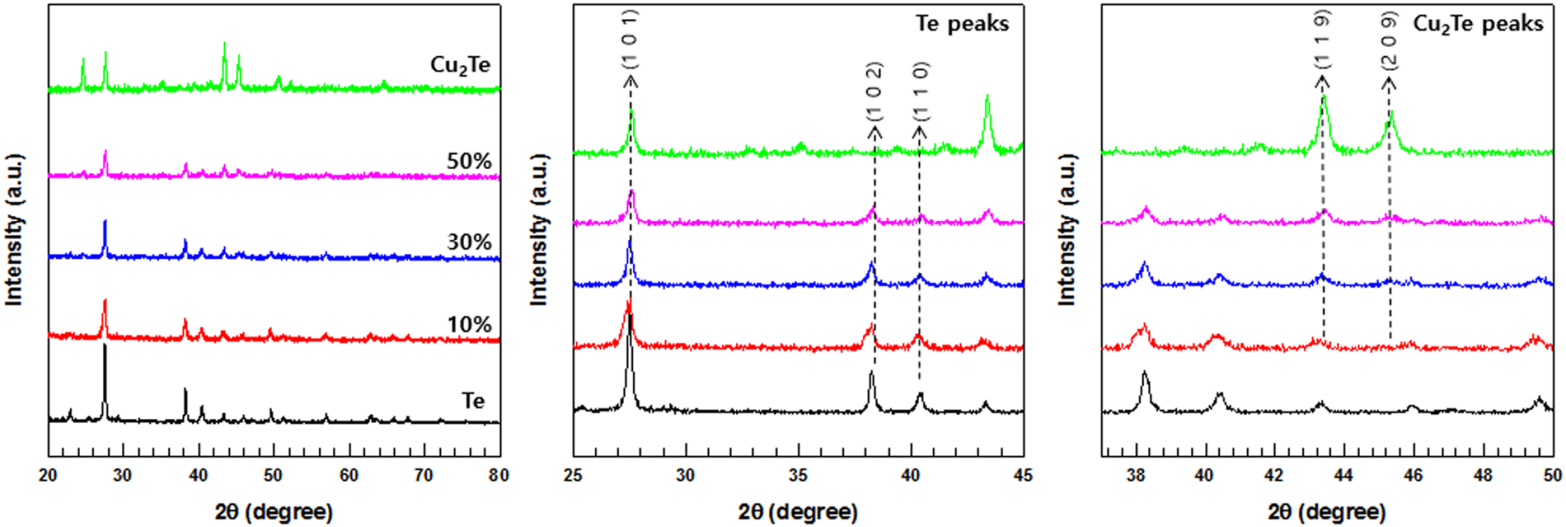
XRD pattern of Te/Cu_2_Te nanorod composites with different Cu_2_Te nanorod contents.

To verify the successful synthesis of the Te/Cu_2_Te composites, XRD analysis was performed on the composites with various Cu_2_Te content. The XRD patterns of the Te/Cu_2_Te composites with different Cu_2_Te content (10, 30, and 50 wt.%) are shown in Fig. 7. The profiles of the composites with low Cu_2_Te content were similar to the XRD peaks of the Te. In contrast, as the Cu_2_Te content increased, the peaks corresponding to Cu_2_Te became more intense. The XRD peaks allowed confirmation that there was good dispersion of Te and Cu_2_Te in the composite samples.

Before measurement of their TE characteristics, the Te/Cu_2_Te nanorod composite samples were compressed into disks with a diameter of 12.7 mm. Pristine Te and Cu_2_Te nanorod disks were also fabricated, all the composite samples were hot-pressed, and then the FE-SEM and EDS analyses were carried out – with the resultant images shown in Figs S1–3.

**Figure 8:**
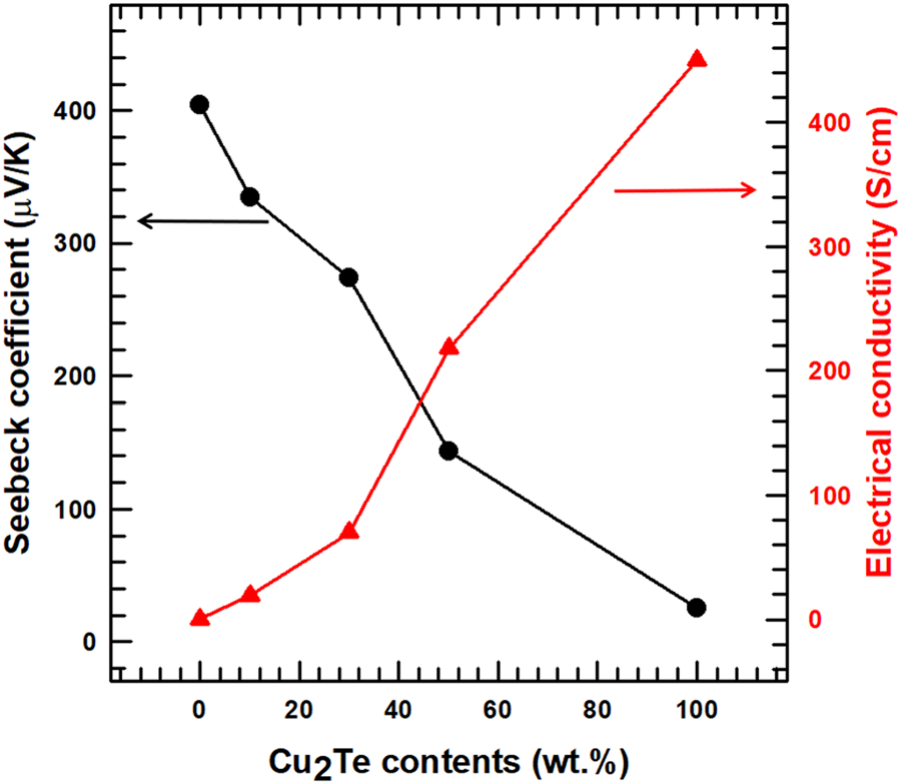
Seebeck coefficient and electrical conductivity of the Te/Cu_2_Te nanorod composite with different Cu_2_Te nanorod content.

The Seebeck coefficient *S* was measured for the composite samples with varied Cu_2_Te content (10, 30, and 50 wt.%) at room temperature. The Seebeck coefficients were determined from the linear relationship between the temperature difference (*ΔT*), and electromotive force (*ΔV*). The measured *S* values of the composite samples are shown in Fig. 8, which also shows the *S* values of the pristine Te and Cu_2_Te nanorods. The *S* values of the two pure nanorods were found to be 404 μV/K and 25 μV/K, which were similar to those in previous reports^7,19^. The Seebeck coefficients of the composite samples showed a decreasing trend with increasing Cu_2_Te contents, which was due to the difference in the *S* values of Te and Cu_2_Te nanorods. Additionally, the *S* values of the Te nanorods, Cu_2_Te nanorods, and Te/Cu_2_Te nanorod composites were all positive, which indicated that Te and Cu_2_Te exhibited p-type semiconductor behavior.

The electrical conductivity of the Te/Cu_2_Te nanorod composite samples with various Cu_2_Te contents (10, 30, and 50 wt.%) are shown in Fig. 8. The electrical conductivity of the two pristine Te and Cu_2_Te nanorod samples were found to be 0.22 S/cm and 454 S/cm, and these values were similar to those in previous reports that analyzed the thermoelectric properties of Te and Cu_2_Te^7,19^. Compared to Te, Cu_2_Te has a much higher electrical conductivity. In contrast to the Seebeck coefficient, as the contents of Cu_2_Te increased, the electrical conductivity of the composite samples also increased. This tendency was due to the difference in electrical conductivity between the two materials.

**Figure 9:**
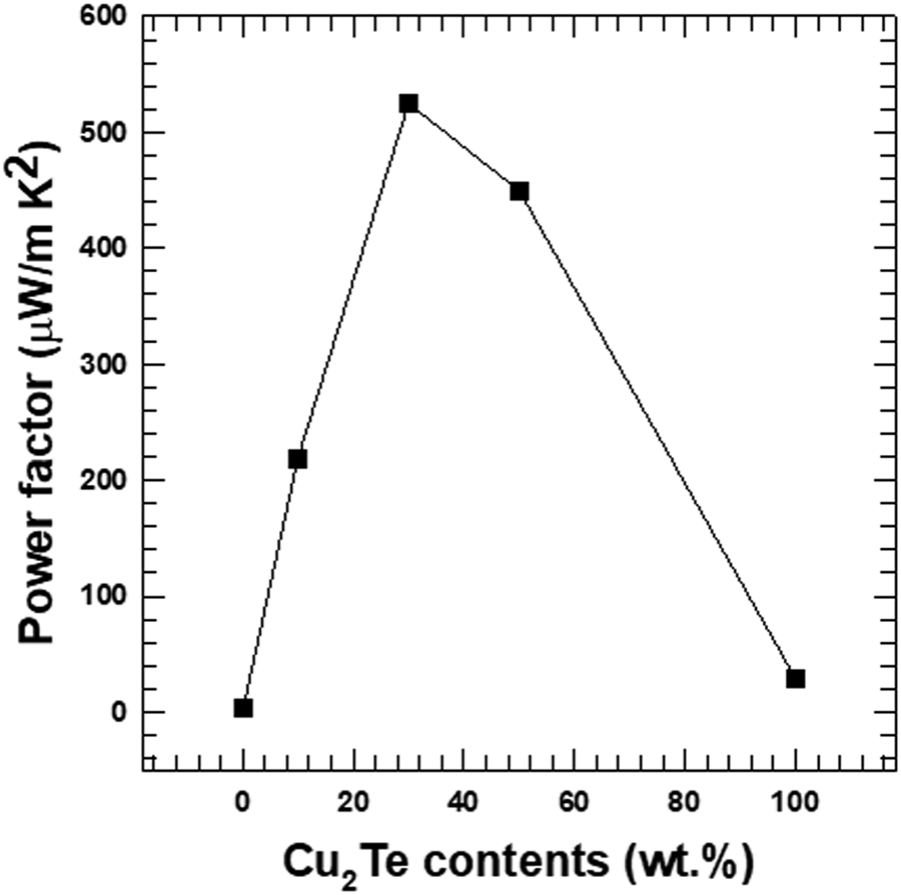
Power factor of the Te/Cu_2_Te nanorod composites with different Cu_2_Te nanorod content.

The thermoelectric power factors of the Te/Cu_2_Te nanorod composites were analyzed, and are shown in Fig. 9. The composite containing 30 wt.% Cu_2_Te nanorods showed the highest power factor (524.6 μW/mK^2^), which was ~145.7 times larger than the PF of the pristine Te nanorods. However, for the Cu_2_Te nanorods with content above 30 wt.%, the power factor showed as decreasing trend, and these changes in the power factors of the samples can be attributed to the reduction of the Seebeck coefficient, and the enhancement of the electrical conductivity.

**Figure 10:**
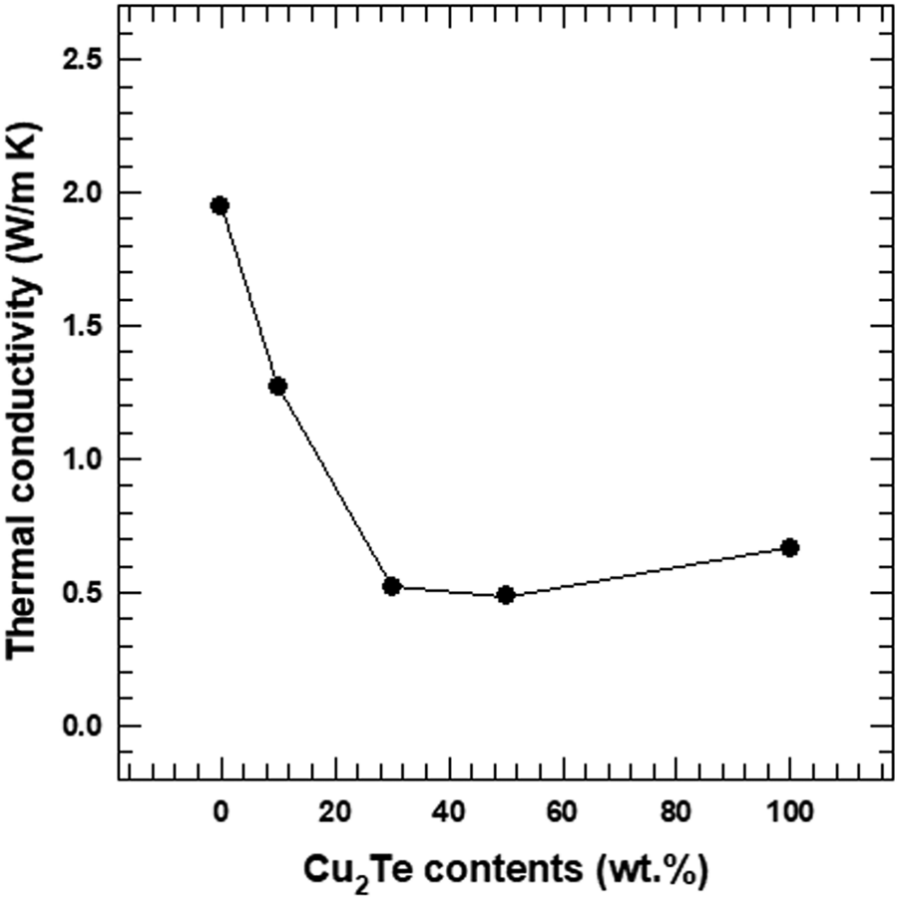
Total thermal conductivity of the Te/Cu_2_Te nanorod composites with different Cu_2_Te nanorod content.

Figure 10 shows the total thermal conductivity of the composites with different Cu_2_Te nanorod content. The total thermal conductivity (*к*) of composite materials is composed of a lattice contribution (*к*_*l*_) from phonons, and an electronic contribution (*к*_*e*_) from the charge carriers (*к* = *к*_*l*_ + *к*_*e*_). The electronic contribution, *к*_*e*_ can be estimated from the Wiedemann-Franz law (*к*_*e*_ = *L·σ·T*), where L is the Lorentz number (L = 2.45 × 10^−8^ WΩ/K^2^)^33–36^. The total thermal conductivity was mainly dependent on the lattice term, *к*_*l*_, due to the relatively small contribution of the electronic term. Therefore, lattice phonon scattering was the key factor that determined the *к* value of the composites. The charge carrier concentration and carrier mobility values of Te/Cu_2_Te composites with different amount of Cu_2_Te are shown in Fig. S4 listed in Table S2.

The *к* values of the pristine Te and Cu_2_Te nanorods (1.94 and 0.67 W/m·K) are also shown in Fig. 10. Specific thermal conductivity parameters for Te/Cu_2_Te nanorod composites, and for the two pristine nanorods, are listed in Table S1. These thermal conductivities were relatively lower than those of the corresponding bulk counterparts reported in a previous study^7,19^. This difference between the *к* values of the bulk and nanorod materials resulted from the decrease in *к*_*l*_, owing to phonon scattering. One-dimensional nanostructures increased phonon scattering, therefore reducing the contribution from *к*_*l*_. Hence, 1D nanomaterials have lower thermal conductivity than their corresponding bulk counterparts, and for this reason, the Te and Cu_2_Te nanorod samples showed lower thermal conductivity than their bulk counterparts. Similar results have been reported in previous studies^26^. The Te/Cu_2_Te nanorod composites showed lower *к* values than the pristine samples.

The findings in this study showed that the incorporation of two nanorod matrices led to the formation of Te/Cu_2_Te nanorod interfaces, which created effective phonon-scattering centers. Due to this, the *к*_*l*_ values of the composites were lower than those of the Te and Cu_2_Te nanorods.

**Figure 11:**
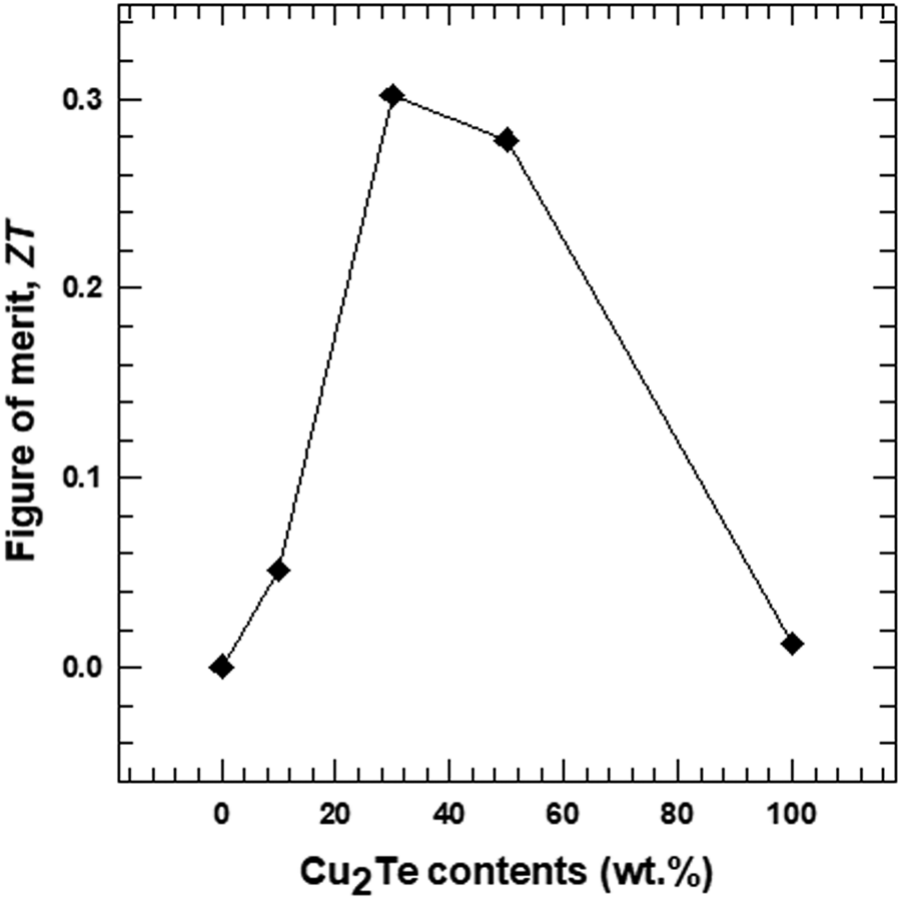
Figure of merit (ZT) of the Te/Cu_2_Te nanorod composite with different Cu_2_Te nanorod content.

The value of *ZT* was determined for the composites, as shown in Fig. 11. The *ZT* values of the Te/Cu_2_Te nanorod composites were higher than those of the two pure nanorods due to their enhanced power factors and reduced *к*. The maximum *ZT* of 0.30 was observed for the 30 wt.% composite sample, which was ~545 times larger than that of the pure Te nanorods.

The Te/Cu_2_Te composites reported in this study showed improved thermoelectric characteristics. The combination of the high electrical conductivity of Cu_2_Te, and the high Seebeck coefficient of Te, was able to enhance the power factor of their composite materials. Additionally, the reduction of thermal conductivity due to the high level of phonon scattering of the nanostructure contributed to the achievement of a high *ZT* value. The present study has shown the synergetic effects of Te and Cu_2_Te materials and highlights the enhanced thermoelectric properties of Te/Cu_2_Te composites.

## Conclusion

Te/Cu_2_Te nanorod composites with various Cu_2_Te contents were successfully fabricated, using a facile wet chemical synthesis and sintering process, and their thermoelectric properties were investigated. During the nanorod synthesis, PVP played an important role in forming the wire-like structure, by reacting with inorganic ions to form chain-shaped intermediates, causing the growth of a one-dimensional nanostructure. The two nanorods were uniformly distributed by ultrasonication and vacuum filtering, providing a well-dispersed solution. XRD, XPS, FE-SEM, and EDS analyses were carried out to confirm the morphology and nanostructure of the nanorod samples.

The main goal of this study was to enhance the electrical conductivity of the Te nanorods. The electrical conductivity of the composite samples showed an increasing trend with increasing Cu_2_Te content, due to the high electrical conductivity of Cu_2_Te. The Seebeck coefficient showed the opposite trend, as a result of the relatively low Seebeck coefficient of Cu_2_Te. As a result, the maximum power factor of the composite sample (524.6 μW·m/K^2^ at 300 K) was observed for the 30 wt.% Cu_2_Te nanorods. This value is ~145.7 times that of the pristine Te nanorods. The composite samples also showed reduced thermal conductivity, due to lattice phonon scattering. The rod-like, 1D nanostructure increased phonon scattering, resulting in decreased lattice thermal conductivity, and total thermal conductivity. The highest thermoelectric figure of merit (*ZT*) was obtained for the 30 wt.% composite sample, and this value was ~545 times that of the pristine Te nanorods. Thus, this study has highlights the synergetic effect of the two nanorods on the thermoelectric properties of the composite.

## Experimental

### Materials

Tellurium (IV) dioxide (TeO_2_, 99%), copper (II) nitrate trihydrate (Cu(NO_3_)_2_·3H_2_O, 99%), L (+)-ascorbic acid (C_6_H_8_O_6_, 99%), hydrazine monohydrate (N_2_H_4_·H_2_O, 80%), and ethylene glycol (EG, C_2_H_6_O_2_, 99.5%) were purchased from Daejung Chemicals & Metals Co (Seoul, Korea). Sodium hydroxide (NaOH, 98%), and PVP, [molecular weight (MW) = ~40,000] were purchased from Sigma Aldrich (St. Louis, USA).

### Synthesis of Te nanorods

First, 2.88 g of TeO_2_ (MW = 159.6), 3.6 g of NaOH (MW = 40), and 1.2 g of PVP were mixed with 120 mL of EG, in the presence of magnetic stirring. Then the solution was heated to 120 °C, after which, 7.35 mL of N_2_H_2_·H_2_O was injected into the mixture. At first, the color of the mixture turned white, which indicated the presence of tellurium dioxide colloids, and then gradually turned dark gray, after the addition of N_2_H_2_·H_2_O. The solution was allowed to react at 120 °C for 90 min. The as-synthesized Te solution was then poured into 10 vol.% N_2_H_2_·H_2_O in de-ionized (DI) water and stirred. Next, it was centrifuged, with the addition of volumetric water, and vacuum-filtered, and the resulting solution was then dried in a vacuum oven, for 24 h at 60 °C.

### Synthesis of Cu_2_Te nanorods

The Cu_2_Te nanorods were synthesized with as-prepared Te nanorods. For this, 1.53 g of the synthesized Te nanorods was dispersed in 60 mL EG. In a separate glass vial, 2.899 g of Cu(NO_3_)_2_·3H_2_O was dissolved in 20 mL EG. The copper precursor solution was then added to the Te nanorod solution, and the mixture was heated to 90 °C, at which point, 12 mL of 1.89 M L (+)-ascorbic acid aqueous solution was injected, to initiate the reaction. The reaction proceeded for two hours under stirring, after which, the solution was washed with DI water, and dried in a vacuum oven.

### Fabrication of Te/Cu_2_Te nanorod composite

The Te/Cu_2_Te nanorod composites were fabricated by ultrasonication of the Te and Cu_2_Te nanorods. Nanorod solutions containing various amounts of Cu_2_Te nanorods (10, 30, and 50 wt.%) were poured into ethanol, and the mixtures were then subjected to ultrasonification for 10 min. The resulting products were washed and filtered, then dried in vacuum oven at 60 °C for 24 h. The resulting composites were ground into a fine powder, and then loaded into a Fe mold and pressed at 200 °C under a pressure of 50 MPa, for 5 min.

### Sample characterization

X-ray photoelectron spectroscopy (XPS, VG-Microtech, ESCA2000) and X-ray diffraction (XRD) (New D8-Advance/Bruker-AXS) at 40 mA and 40 kV using a Cu Kα radiation (0.154056 nm) source and a scan rate of 1°/s in the 2*θ* range of 5 to 70° were employed to characterize the crystal structure of the materials. Field-emission scanning microscopy (FE-SEM, SIGMA) was used to determine the morphology and microstructure of the materials. Elemental maps of the sample were analyzed using energy-dispersive X-ray diffraction (EDS, NORAN system 7, Thermo Scientific).

The Seebeck coefficient, *S*, was then calculated as the ratio between *ΔV* and *ΔT*, given as *S* = *ΔV/ΔT*. The value was calculated from the slope of the line representing the linear relationship between the thermal electromotive force (*ΔV*), and the temperature difference (*ΔT*), between the two end points of the composite pellets. A four-point probe with a source meter (Keithley 2400) was used to measure the electrical conductivity, and a digital micrometer was used to measure the thickness of the sample. Charge carrier concentration and carrier mobility of the composite were determined by conducting Hall-effect measurements using a Van der Pauw four-point probe configuration (HMS-3000, Ecopia). The thermal conductivity of the sample was calculated using the equation *κ* = *α·ρ·C*_*p*_, where *α*, *ρ*, and *C*_*p*_ are the thermal diffusivity, bulk density, and specific heat of the material, respectively. The xenon flash method was conducted using NETZSCH, LFA 447 Nanoflash instrument to evaluate *α*, whereas *C*_*p*_ was measured by applying differential scanning calorimetry (DSC) (DSC 131 EVO, Setaram Instrumentation).

## Electronic supplementary material

Supplementary information

## Data Availability

All data generated or analyzed during this study are included in this paper. Raw datasets are available from the corresponding author, upon receipt of a reasonable request.
