# Supplementary material for: Facile fabrication of one-dimensional Te/Cu2Te nanorod composites with improved thermoelectric power factor and low thermal conductivity
Source: Sci Rep. 2018 Dec 24;8:18082. doi: 10.1038/s41598-018-35713-9 (PMC6305380; doi:10.1038/s41598-018-35713-9)
Supplement: Supplementary file 1 — Supplementary information [file 41598_2018_35713_MOESM1_ESM.docx]

**Supplementary Information:**

**Facile fabrication of one-dimensional Te/Cu_2_Te nanorod composites with improved thermoelectric power factor and low thermal conductivity.**

Dabin Park, Hyun Ju, Taeseob Oh and Jooheon Kim*

School of Chemical Engineering & Materials Science,

Chung-Ang University, Seoul 06974, Republic of Korea

*Corresponding author: jooheonkim@cau.ac.kr (J. Kim)

Supporting Information Contents:

1. Figures

2. Tables

**1. Figures**

**
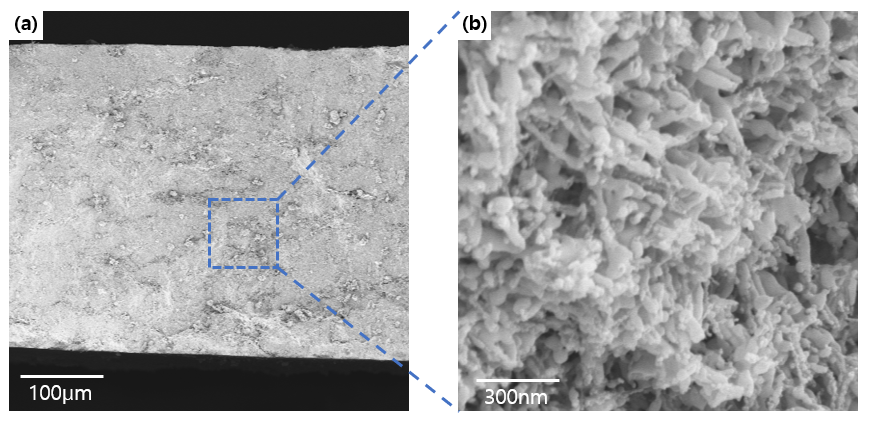
**

Figure. S1. (a) Low and (b) high-magnification FE-SEM images of hot-pressed Te/Cu_2_Te nanorod composites.

**
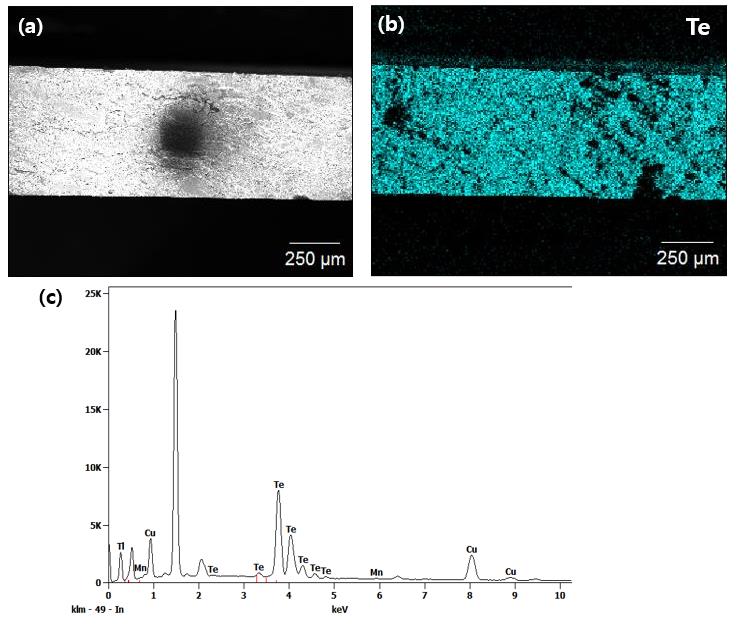
**

Figure. S2. FE-SEM images with the corresponding EDS elements mapping (a-b) and EDS spectra of Te nanorods (c)


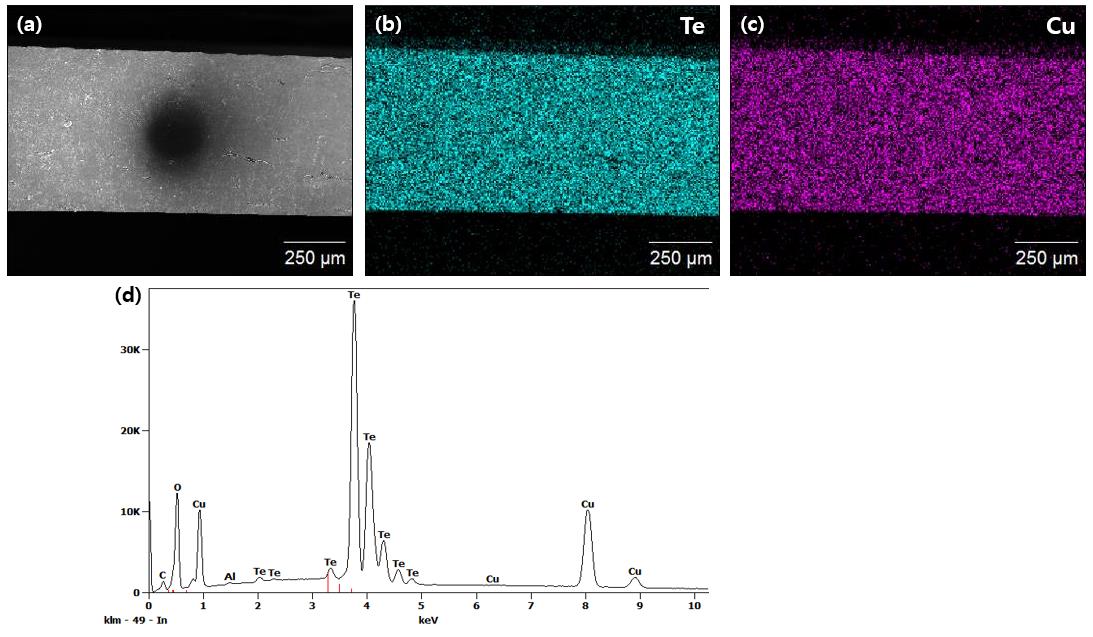


Figure. S3. FE-SEM images with the corresponding EDS elements mapping (a-c) and EDS spectra of Cu_2_Te nanorods (d)


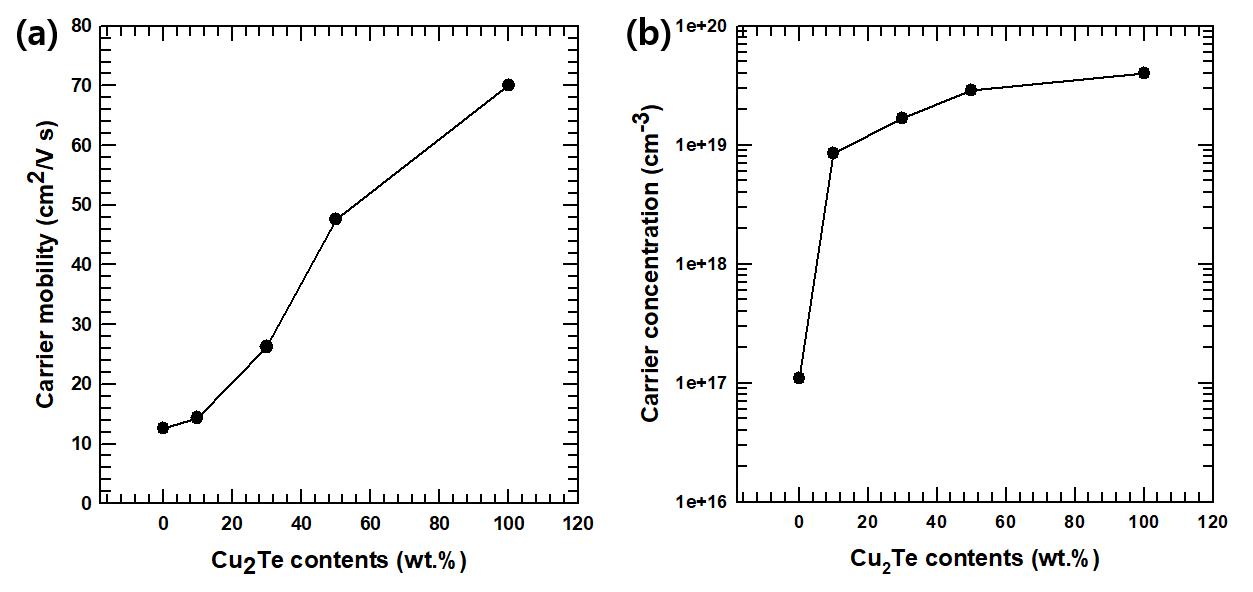


Figure. S4. (a) carrier mobility, and (b) carrier concentration of Te/Cu_2_Te nanorod composites with various Cu_2_Te contents

**2. Tables**

|  | *ρ*  (g/cm^3^) | *κ*  (W/m·K) | *σ*  (S/cm) | *Ref.* |
| --- | --- | --- | --- | --- |
| Te nanorod | 5.791 | 1.94 | 0.22 | This study |
| 10 wt.% Cu_2_Te | 6.512 | 1.27 | 19.43 | This study |
| 30 wt.% Cu_2_Te | 6.616 | 0.52 | 69.95 | This study |
| 50 wt.% Cu_2_Te | 6.755 | 0.48 | 217.94 | This study |
| Cu_2_Te nanorod | 7.015 | 0.67 | 454.1 | This study |
| Bulk Te | 6.4 | 4~12 | 1.5~5 | 1, 14 |
| Bulk Cu_2_Te | 7.15 | 1~1.3 | 600~1200 | 35, 36 |

Figure. S3. Table S1. Density, thermal conductivity, and electrical conductivity of different types of Te, Cu_2_Te, and Te/Cu_2_Te nanorod composites.
